# 3D-Dixon cardiac magnetic resonance detects an increased epicardial fat volume in hypertensive men with myocardial infarction

**DOI:** 10.1186/1532-429X-18-S1-O4

**Published:** 2016-01-27

**Authors:** Rami Homsi, Alois Martin Sprinkart, Juergen Gieseke, Seyrani Yuecel, Darius Dabir, Julian A Luetkens, Christian Marx, Daniel Kuetting, Hans H Schild, Daniel K Thomas

**Affiliations:** 1 grid.15090.3d000000008786803XRadiology, University Hospital Bonn, Bonn, Germany; 2 grid.5570.7000000040490981XInstitute of Medical Engineering, Ruhr-University, Bochum, Germany; 3 Philips Healthcare, Bonn, Netherlands; 4 Cardiology, Gemeinschaftskrankenhaus Bonn, Bonn, Germany

## Background

Increased epicardial adipose tissue is associated with cardiovascular risk and disease, such as hypertension. Hypertensive patients are at greater risk for the development of myocardial infarction. Following validation of an ECG- and respiratory triggered 3D-Dixon pulse sequence we investigated for the first time epi- and pericardial fat volumes (EFV, PFV) in hypertensive men with and without myocardial infarction (MI).

## Methods

**Figure 1:**
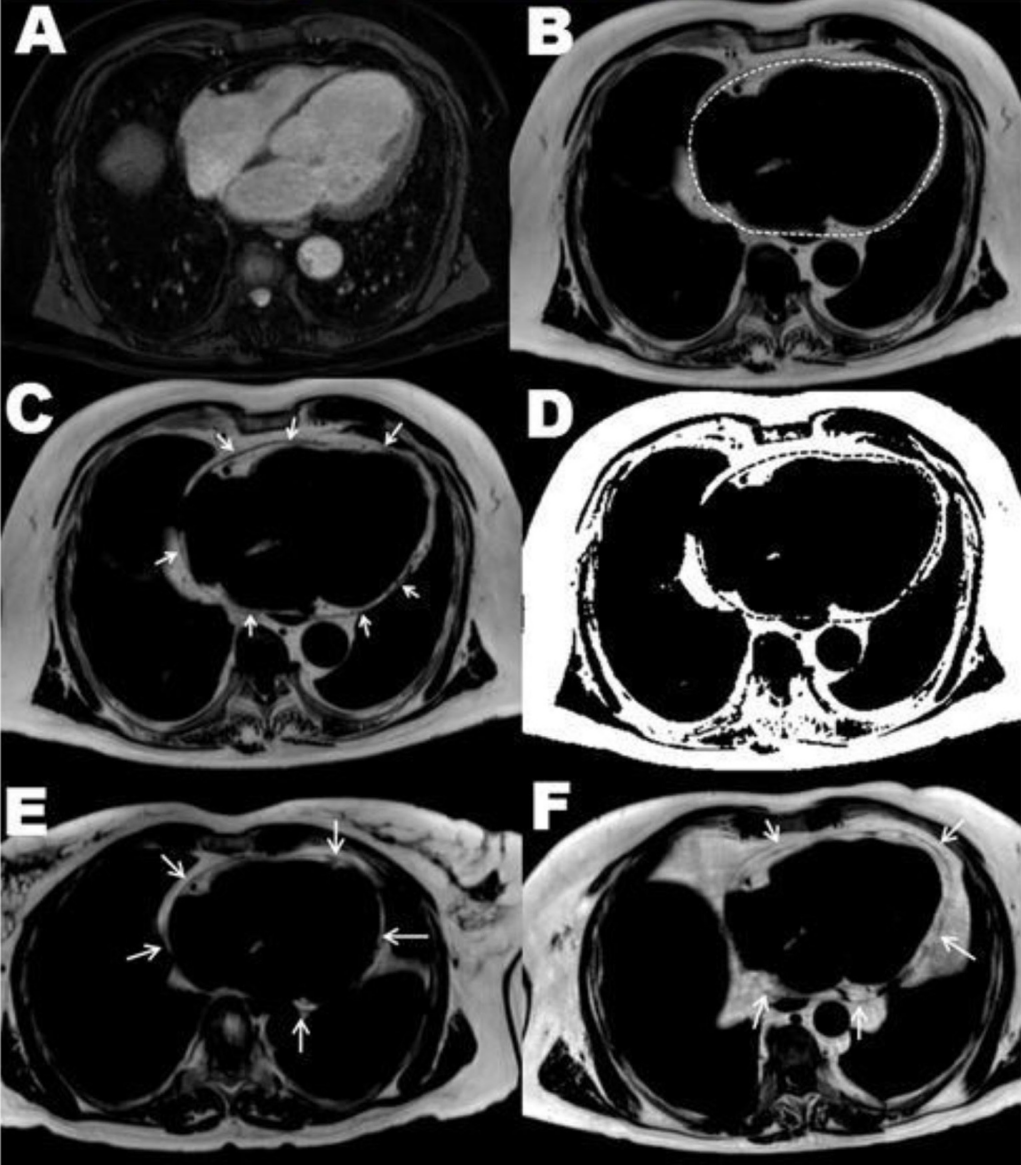
**(A-D) show the Dixon image analysis of epi-and pericardial fat in a 61 year old hypertensive male patient with known 1-vessel disease of the left anterior descendent artery, but without myocardial infarction: (A) reconstructed water only image**. (B) and (C) reconstructed fat only image with the pericardial outlines. (D) computed fat-fraction map based on fat- and water-only images (with the transferred regions of interest). (E and F) show fat only images of a 67 year old health man (E) and of a 64 year old hypertensive man with known 3-vessel disease and myocardial infarction (F) with the arrows pointing at the pericardial outlines.

55 hypertensive men (mean age 63.02 ± 10.73 years [y]) with and without coronary artery disease (CAD) and ten healthy controls were (mean age 59.00 ± 8.41 y) underwent a comprehensive cardiomagentic resonance (CMR) examination on a 1.5 Tesla MR system (Ingenia, Philips). EFV was assessed using a 3D transversal ECG- and respiratory navigator gated mDixon-sequence (3D-Dixon). Fat only images were reconstructed online at the scanner, and the segmentation of fat volumes was performed based on fat fraction maps (figure 1) using in-house software written in MATLAB (The MathWorks, Inc., Natick, MA). EFV and PFV were normalized to the body surface area.

## Results

The healthy control group had significantly lower EFV and PFV than hypertensive men (52.98 ± 19.81 and 115.50 ± 53.57 versus 81.8 ± 33.90 and 194.86 ± 83.51; P < 0.05). EFV and PFV were also lower compared to patients with only hypertension and no CAD (n = 18; EFV 74.53 ± 26.40; PFV 174.60 ± 65.70; P < 0.05). Hypertensive men with MI (22 men, mean age 61.55 ± 10.50 y; EFV 94.14 ± 43.16, PFV 224.26 ± 100.79) had significantly higher fat volumes than hypertensive men without MI (33 men, mean age 63.17 ± 10.93 y; EFV 73.57 ± 23.27, PFV 175.26 ± 63.07; [P < 0.05, each]). At the same time there was no relationship with the presence or severity of CAD in patients without MI (n = 15; mean age 69.47 ± 10.70 y). There were no significant differences in age, BMI or heart rate between the groups.

## Conclusions

3D-Dixon measurements revealed significantly higher epicardial fat volumes in hypertensive men with myocardial infarction compared to hypertensive men without myocardial infarction. This finding underscores the role of fatty tissue as an endocrine and metabolically active organ. Noninvasive CMR-based whole volume measurement of epicardial fat may play a relevant future role in cardiovascular risk stratification and disease management.

